# Is creatine a CNS neurotransmitter?

**DOI:** 10.7554/eLife.91824

**Published:** 2023-10-16

**Authors:** Bhagaban Mallik, C Andrew Frank

**Affiliations:** 1 Department of Anatomy and Cell Biology, University of Iowa Iowa City United States

**Keywords:** creatine, inhibitory neurotransmission, synaptic vesicles, astrocytes, SLC6A8, AGAT, Mouse

## Abstract

A range of experiments suggests that creatine, a molecule known for recycling ATP in muscle and brain tissue, may also function as a neurotransmitter in the central nervous system.

**Related research article** Bian X, Zhu J, Jia X, Liang W, Yu S, Li Z, Zhang W, Rao Y. 2023. Evidence suggesting creatine as a new central neurotransmitter: presence in synaptic vesicles, release upon stimulation, effects on cortical neurons and uptake into synaptosomes and synaptic vesicles. *eLife* **12**:RP89317. doi: 10.7554/eLife.89317.

Communication in the nervous system relies on neurons transmitting signals to target cells. This process is facilitated by various chemicals, including neurotransmitters, neuromodulators and neuropeptides (Langley, 1905; Benfenati and Agnati, 1991; Lovinger, 2008). Identifying a new neurotransmitter is no small feat: such a discovery requires extensive investigation and validation, and it might take decades to integrate the collective contributions of many different research groups (Hökfelt, 2010; Contestabile, 2011). Moreover, neurotransmitters that act in peripheral tissues, like muscle, are easier to identify than those that act on the central nervous system (CNS). It is likely that many CNS neurotransmitters have not yet been identified (Curtis et al., 1959; Carlsson et al., 1962; Curtis and Watkins, 1963; Björklund et al., 1968; Andersson, 2000).

To be classified as a neurotransmitter, a molecule should meet several criteria: it needs to be stored within a synaptic vesicle in a neuron and be released upon neuronal stimulation; it needs to act upon a postsynaptic receptor; and afterwards, it needs to be removed or retrieved from the synaptic cleft (Kandel et al., 2021; Purves, 2018; Radian et al., 1986; Pacholczyk et al., 1991). Curiously, several molecules widely accepted as neurotransmitters only meet some, rather than all, of these criteria (Curtis and Watkins, 1960; Felix and Künzle, 1974). Accurately detecting candidate molecules within synaptic vesicles could represent a significant step towards identifying neurotransmitters with a higher certainty.

Now, in *eLife*, Yi Rao and colleagues at Peking University and other research centers in Beijing – including Xiling Bian, Jiemin Zhu and Xiaobo Jia as joint first authors – report data that potentially uncovers a new neurotransmitter within the mammalian brain (Bian et al., 2023). The researchers used a combination of mass spectrometry, genetics, biochemistry, immunostaining, electrophysiology, and electron microscopy to support this conclusion.

Starting with purified synaptic vesicles (SV) from mouse brains, Bian et al. detected several well-known neurotransmitters in their samples, as well as creatine. The levels of SV creatine were higher than those of other known neurotransmitters, such as acetylcholine and serotonin, but lower than glutamate and gamma-aminobutyric acid (GABA). It has been known for decades that creatine is involved in recycling ATP in both muscle and brain tissue (Wyss and Kaddurah-Daouk, 2000; Brosnan and Brosnan, 2007; Wallimann et al., 2011). And more recently, researchers have suggested that it might also have additional roles in brain function (Ohtsuki et al., 2002; Braissant et al., 2011).

The next step was to dissect the molecular mechanism of creatine function in neurons. Bian et al. demonstrated that creatine was released from stimulated coronal brain slices. Interestingly, creatine release was reduced in slices from mice lacking either the gene that codes for an enzyme called AGAT (which is necessary for creatine production), or the gene that codes for the SLC6A8 creatine transporter. Importantly, the Bian et al. also observed that creatine has an inhibitory effect on a subset of neurons. They also found that SLC6A8 can move creatine into synaptosomes (isolated synaptic structures that contain a machine that helps release neurotransmitters and large numbers of synaptic vesicles). Collectively, these results are consistent with creatine acting like a neurotransmitter and with AGAT and SLC6A8 supporting that function (Figure 1).

**Figure 1.**
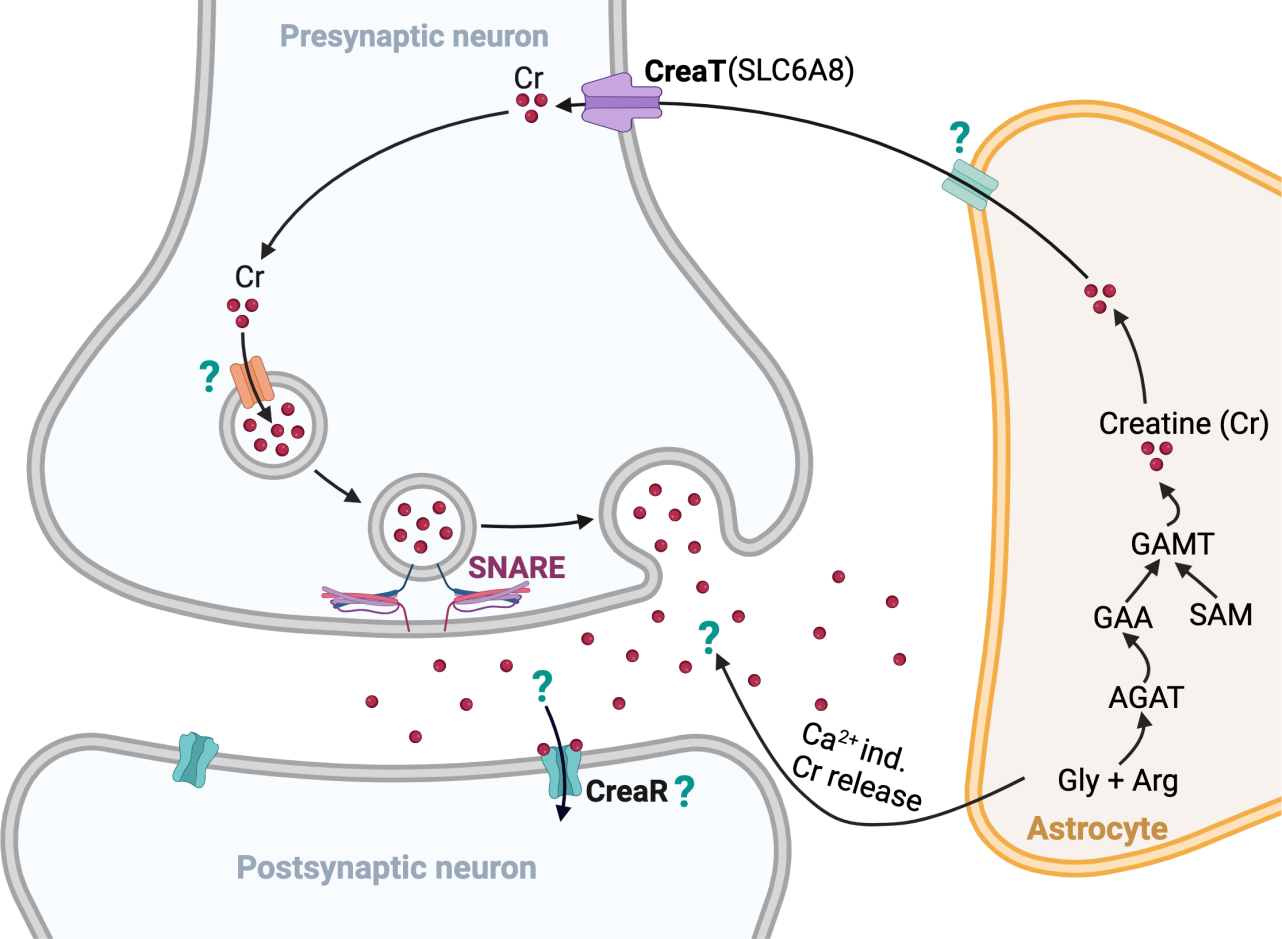
Schematic illustration of the synthesis, transport and release of creatine in neurons. A subtype of glial cell, known as an astrocyte (orange), likely synthesizes creatine molecules (red dots) through a process involving the amino acids glycine (Gly) and arginine (Arg), the enzymes AGAT and GAMT, and other compounds (GAA and SAM; Roschel et al., 2021). It is likely that the creatine molecules are then transported into a synaptic vesicle located in a presynaptic neuron that expresses a creatine transporter (CreaT) called SLC6A8. SNARE proteins (string-like structures) then mediate the release of the vesicles containing the creatine molecules into the synaptic cleft in a calcium-dependent manner, and the creatine molecules go on to bind to an as-yet unidentified creatine receptor (CreaR) on the postsynaptic neuron. It is possible that creatine molecules are also released directly by the astrocytes into the synaptic cleft between the neurons in a calcium-independent manner. AGAT: L-arginine: glycine amidinotransferase; GAA: guanidinoacetate; GAMT: guanidinoacetate methyltransferase; SAM: S-adenosylmethionine.

Image created with BioRender.com.

The work by Bian et al. goes beyond previous studies, which posited that creatine could have neurotransmitter-like properties (Almeida et al., 2006; Peral et al., 2010). Nevertheless, questions remain for future work. Most notably, Bian et al. did not identify a specific postsynaptic receptor for creatine. The researchers speculate that there might be a metabotropic receptor (or receptors) for creatine (Figure 1). Another mystery is that most of the creatine release after high potassium stimulation occurs when there is no extracellular calcium present. This is not consistent with a neurotransmitter role for that portion of the release. Bian et al. speculate that astrocytes might be responsible because astrocytes contain high levels of an enzyme called GAMT, which is involved in the production of creatine. If this idea were correct, then astrocytic creatine could potentially serve a neuromodulatory role. One final puzzle is that AGAT and SLC6A8 are found in different cells in the brain. So if creatine were a neurotransmitter, it suggests a complex model of creatine being synthesized in one cell type and subsequently then transported to another cell type for release (Figure 1).

In summary, Bian et al. report that creatine is a possible neurotransmitter in the central nervous system and that it meets several textbook criteria for a neurotransmitter (Kandel et al., 2021; Purves, 2018). This is a potentially groundbreaking finding that could have implications for understanding brain function and neurotransmission. It may also open new areas of understanding Creatine Transporter Deficiency, which manifests as a collection of intellectual disabilities, language delays and other neurological disorders that are associated with defective SLC6A8 (Salomons et al., 2003).
